# Current guidelines for the management of rectal cancer patients: a review of recent advances and strategies

**DOI:** 10.1590/1806-9282.2024S112

**Published:** 2024-06-07

**Authors:** Carlos Augusto Real Martinez, Fábio Guilherme Campos

**Affiliations:** 1 Universidade Estadual de Campinas, Department of Surgical – Campinas (SP), Brazil.; 2 Universidade São Francisco, Medical Course – Bragança Paulista (SP), Brazil.; 3 Universidade de São Paulo, Medical School, Clinical Hospital, Department of Gastroenterology, Division of Colorectal Surgery – São Paulo (SP), Brazil.

## INTRODUCTION

Colorectal cancer (CRC) is a common and lethal disease, the third most common cancer diagnosed in both men and women in the United States^1^. The American Cancer Society's estimates are 106,970 new cases of colon cancer and 46,050 new cases of rectal cancer (RC) for 2023^2^.

Despite this, general rates dropped by about 1% each year from 2011 to 2019, probably due to the use of screening colonoscopy^3–5^. However, this decrease occurred in the older population, as in the population under 50 years, the rates increased by 1–2% a year since mid-1990s^1^. In Brazil, the National Cancer Institute (INCA) estimates 45,630 new CRC cases will be diagnosed annually in 2023–2025^4^.

Colorectal cancer results from the interaction of genetic predisposition and environmental risk factors, but increasing age remains the most important risk factor. In this setting, CRC familial history, personal history of adenomas or inflammatory bowel disease, and inherited syndromes should always be evaluated^5,6^. Simultaneously, tobacco, alcohol use, obesity, lack of physical activity, and unhealthy lifestyle choices, such as a diet high in processed meats and low in fruits and vegetables, sedentary behavior, obesity, smoking, and excessive alcohol consumption, have been associated with increased risk^6^.

Colorectal cancer development occurs from genetic defects (mutations), inherited or acquired^7,8^. Also, chemical, physical, or biological agents in the intestinal lumen may cause colonocyte DNA damage and form cell clones with neoplastic cell attributes. A better understanding of the mechanisms by which a normal epithelium of the colon transforms into an adenoma and, subsequently, into an invasive carcinoma has become possible with the clarification of the adenoma-carcinoma sequence^7,8^.

At the molecular level, CRC is a heterogeneous disease due to at least three major molecular tumorigenesis pathways. The most common (85%) is classical chromosomal instability (CIN). These mechanisms are typically associated with mutations in oncogenes or tumor suppressor genes such as adenomatous polyposis coli (APC) and others that regulate cell proliferation^8^.

The microsatellite instability (MSI) pathway is caused by a deficiency of the DNA mismatch repair gene^9,10^. And the serrated pathway is responsible for approximately 20–30% CRC cases. There may be some overlap between these mechanisms, which explains the different molecular features existing in CCR^9,10^.

In 2012, the Cancer Genome Atlas Network (CGAN) classified CRC into four subtypes with distinct molecular, biological, and clinical characteristics: CMS1 (microsatellite instability immune), CMS2 (canonical), CMS3 (metabolic), and CMS4 (mesenchymal)^11^.

## CLINICAL PRESENTATION AND INITIAL EVALUATION

Rectal cancer represents around 30% of all CRC tumors. Symptoms like hematochezia, tenesmus, and mucous discharge always suggest a rectal location. Other complaints are anemia, abdominal pain, changes in bowel habits, and weight loss^12,13^.

Initial evaluation is made by detailed anamnesis, digital rectal examination, endoscopic assessment, tissue biopsy, and serum carcinoembryonic antigen (CEA). Colonoscopy may identify polyps or synchronous tumors upstream of the primary lesion located in the rectum.

Preliminary information from proctological and gynecological examinations is crucial, such as distance from the anal verge and vaginal infiltration. The finding of ascites, hepatomegaly, inguinal nodes, and severe malnutrition may raise the possibility of metastatic disease.

Local and distant staging is achieved with chest and abdominal computed tomography and pelvic magnetic resonance imaging (MRI). Depending on specific findings, a transrectal ultrasound or a positron emission tomography-computed tomography (PET-CT) scan may add some information.

Extracted information from an MRI may help to evaluate the possibility of achieving free radial and distal margins after surgery, the tumor's relation to the mesorectal fascia, peritoneal reflection, and anorectal muscular ring. Other data include invasion of the rectal wall, mesorectum, and adjacent organs or structures (T status). Likewise, the number and appearance of lymph nodes, the presence of tumor deposits in the mesorectum (N status), the presence or absence of vascular invasion, and the enlargement of the lymph nodes of the lateral pelvic wall must be described.

**Figure 1:**
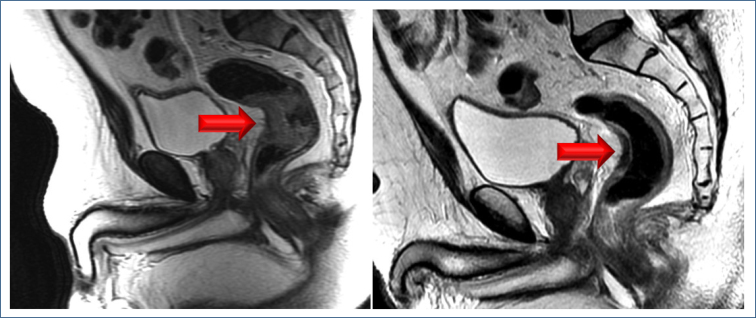
(A) Middle rectal tumor before neoadjuvant chemoradiotherapy (red arrow). (B) Good response after neoadjuvant chemoradiotherapy (red arrow). Courtesy of the Department of Radiology at UNICAMP (Prof. Daniel Lahan).

After neoadjuvant treatment, a re-evaluation with MRI, digital rectal examination, and endoscopy in accessible lesions will select bad and good responders (Figure 1)^14^. The selection of complete responders may allow organ preservation with a watch and wait (W&W) strategy and long-term surveillance. All other patients should deserve surgical treatment.

More recently, the colorectal community in Western Europe has driven attention to the importance of lateral pelvic lymph node involvement, mainly in distal rectal tumors^15^. Patients with locally advanced rectal cancers (LARC) who present enlarged lateral pelvic lymph nodes (>5 mm in their shortest axis) after neoadjuvant chemoradiotherapy should undergo lateral pelvic lymphadenectomy.

## THE ROLE OF MULTIMODALITY MANAGEMENT

Before the 1980s, surgical resection was considered the best option for all tumor stages. However, high recurrence rates led to the evaluation of neoadjuvant chemoradiation as an integral part of RC treatment before total mesorectal excision (TME)^16^. Neoadjuvant chemotherapy in stages II (T3 or T4 node-negative) and III (node-positive) patients aims to reduce local and distant recurrence rates, besides having no survival benefits.

Nowadays, a multidisciplinary team (the tumor board) composed of a radiotherapist, oncologist, and colorectal surgeon should discuss together the best combination of chemoradiation protocol and surgery. Attempts to design new therapeutic strategies included different drug combinations, modifications in the sequence and duration of chemotherapy protocols, dose and radiotherapy duration, and the time interval between neoadjuvancy and surgery. Simultaneously, it was possible to gradually increase the number of patients treated with nonoperative management (NOM), an option that was introduced by Habr-Gama et al.^17^ in Brazil. In this setting, patients are not referred for immediate surgery and are put under close surveillance^17^. Published results from the International W&W Database (IWWD) have corroborated the safety of the NOM strategy, and the number of patients undergoing NOM has progressively increased^18^.

Another strategy called total neoadjuvant therapy (TNT) was designed to offer all chemotherapy before surgery, aiming to ensure that a greater fraction of patients would complete all chemotherapy regimens (induction or consolidation chemotherapy) before chemotherapy. A series of phase III randomized multicentric studies have evaluated different TNT regimens in LARC patients, the so-called RAPIDO, PRODIGE-23, and OPRA trials^19–21^.

In the first two, it was demonstrated that patients undergoing the TNT protocol had lower rates of distant recurrence, despite no gain in overall survival (OS)^19,20^. In the OPRA study, a prospective randomized phase II trial assessed the outcomes of patients with stage II or III LARC treated with two different protocols^21^. The study concluded that the introduction of different TNT protocols allowed organ preservation in half of them without an apparent detriment to survival^21^.

## THE BASIS FOR MODERN SURGICAL TREATMENT

**Figure 2:**
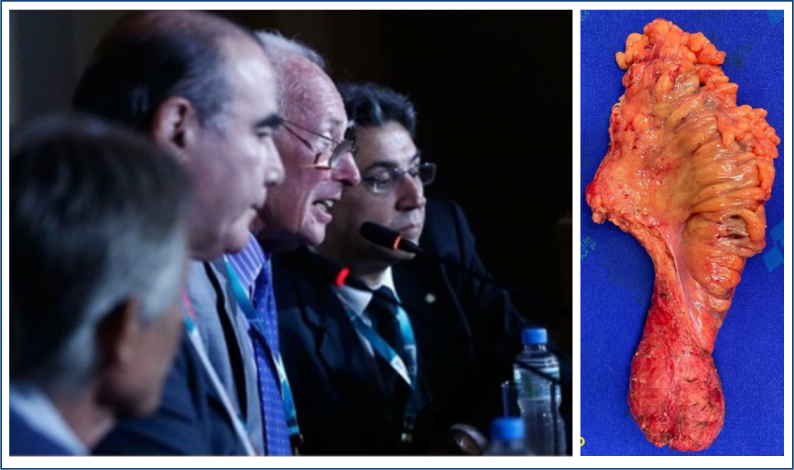
Dr. Richard Heald between the authors Campos FG (right) and Martinez CA (left) during a visit to Brazil some years ago. On the right, a surgical specimen of rectal cancer with total mesorectal excision. Right: Courtesy: CARM, Left: Courtesy: Department of Surgery at UNICAMP.

Rectal resection was historically studied and designed by the famous English surgeon William Ernest Miles (Figure 2), who published his seminal paper in 1908 and initiated the era of radical resections to treat RC^16,22^.

Surgery for RC involves complex decisions and great challenges for colorectal surgeons. Primary lesions are managed with variable endoscopic, endoanal, or surgical procedures depending on the surgeon's experience, patients', and tumor features. Treatment may be performed with endoscopic or surgical techniques.

## MANAGEMENT OF LOCALIZED RECTAL CANCER

Local resection of RC performed by endoscopic or surgical approaches may be offered to selected tumors and well-informed patients agreeing with close surveillance. Patients with T0-1N0 lesions smaller than 3 cm and clinically mobile will benefit from this approach, although recurrence rates (7–21%) may be higher than radical resection. The presence of favorable histologic features in a pedunculated polyp will not require further surgery.

As well, those considered unfit for surgical radical resection may also be candidates. Features such as muscularis propria invasion (T2 tumors), poor histological grade, lymph nodes, vascular or perineal invasion, and flat or depressed morphology are deemed high-risk factors for this type of procedure.

Submucosal invasion greater than 1000 micrometers may lead to a 12% nodal involvement rate. Similarly, surgical resection may be indicated if patients treated with endoscopic resection exhibit fragmented or not assessable margins. The same idea is not applied to T2 lesions, where recurrence rates may achieve 26–47% in patients^23^. In those presenting an almost complete response after neoadjuvant treatment, endoanal local excision may also be recommended, despite wound complications in a rectum previously irradiated^24^.

## TRANSABDOMINAL RESECTION

A transabdominal resection may be required to treat LARC in the upper, middle, or low rectum. Surgery must remove the tumor-bearing bowel with adequate margins while preserving functions.

The introduction of TME represented a great technical advance that significantly reduced local recurrence rates. Technical details were designed and disseminated among colorectal surgeons by Dr. Richard Heald (Figure 2).

A 1-cm distal margin is generally adequate for well- or moderately differentiated tumors. After TME, a temporary deviation with an ileostomy is advisable to protect the anastomosis. In cases with direct involvement of the anal sphincters or levator muscles, an abdominoperineal excision of the rectum (APR) with a definitive colostomy will be necessary. Prophylactic dissection of lateral pelvic lymph nodes is not advisable, but this approach is recommended when lateral lymph node enlargement is detected in restaging MRI.

## MINIMALLY INVASIVE TECHNIQUES

In recent decades, the introduction of minimally invasive surgery (MIS) in RC surgery has provided excellent outcomes^25^. Both laparoscopic and robotic approaches seem to have excellent short- and long-term results when compared to conventional access. Evidence suggests numerous MIS advantages, besides greater costs^26^. A meta-analysis of randomized clinical trials comparing laparoscopic and open rectal resection for cancer was performed by analyzing a total of 26 end points^27^. They demonstrated that laparoscopic surgery for RC was associated with a statistically significant reduction in intraoperative blood loss and the number of blood transfusions, an earlier resuming of a solid diet, a return of bowel function, and a shorter duration of hospital stay. Laparoscopy also reduced post-operative abdominal bleeding, late adhesion obstruction, and morbidity. No differences were found in terms of intraoperative and late oncological outcomes.

A recent meta-analysis compared the long-term oncologic outcomes of laparoscopic and open surgery^28^. The 5-year estimated disease-free survival (DFS) rates were 72.2% for the laparoscopic group and 70.1% for the open surgery group, with 5-year estimated OS rates of 76.2 and 72.7%, respectively. The OS was significantly better in the laparoscopic group. The authors concluded that a similar DFS but a significantly better OS were found for patients who had undergone laparoscopic surgery.

Robot-assisted colorectal surgery is an evolving field suitable for transabdominal, trans-anal, and endoluminal approaches and encompasses many surgical techniques, including dissection, resection, and anastomosis. It is particularly advantageous in confined spaces such as the low rectum and endoluminal areas. While robotic surgery has great potential for improving outcomes, its' possible disadvantages over traditional laparoscopy and open surgery are still being debated^29^. Due to the advantages of greater freedom of movement, increased three-dimensional (3D) vision, better ergonomics, and a static camera, robotic surgery has provided greater surgical quality in difficult situations, such as inferior rectal tumors.

The ability to expose and separate fine tissues provides better dissection of embryological planes and drastically reduces damage to pelvic nerves and blood vessels by providing a clear view and identification of small nerves, thus protecting urinary and sexual functions. The robotic access allows for easier access to the lower rectum, particularly in obese men and those with a narrow pelvis. Studies confirm that the robotic approach in obese patients resulted in a shorter length of stay and a lower 30-day readmission rate, but longer operative time when compared to laparoscopic surgery. Robotic rectal surgery in the obese may be associated with a quicker postoperative recovery and a reduced morbidity profile^30^.

In conclusion, robotic surgery is a rapidly evolving field that offers many benefits over traditional surgical methods. Robotic platforms have enabled surgeons to perform procedures with greater precision, dexterity, and flexibility. Additionally, robotic surgery has reduced pain and recovery time, leading to shorter hospital stays and improved clinical outcomes. Despite its advantages, robotic surgery still has limitations, such as undefined long-term oncologic outcomes, the need for specialized training, incompatible instruments, higher costs, and the lack of haptic feedback. However, ongoing technological advancements and studies are addressing these limitations and opening up new possibilities for the future of surgical robotics.

## CONCLUSION

Rectal cancer is a complex and challenging disease in which oncological outcomes depend on accurate diagnosis, multidisciplinary management, and specialized surgery. Treatment should typically incorporate a tumor board discussion to define the best therapeutic option to achieve good results, and therefore it should be preferably planned in specialized centers.
